# Vertical policy coordination of COVID-19 testing in Sweden: an analysis of policy-specific demands and institutional barriers

**DOI:** 10.1108/JHOM-09-2022-0278

**Published:** 2024-03-11

**Authors:** Anna Hallberg, Ulrika Winblad, Mio Fredriksson

**Affiliations:** Department of Public Health and Caring Sciences, Uppsala University, Uppsala, Sweden

**Keywords:** COVID-19 testing, Decentralization, Vertical policy coordination, Sweden, Healthcare governance

## Abstract

**Purpose:**

The build-up of large-scale COVID-19 testing required an unprecedented effort of coordination within decentralized healthcare systems around the world. The aim of the study was to elucidate the challenges of vertical policy coordination between non-political actors at the national and regional levels regarding this policy issue, using Sweden as our case.

**Design/methodology/approach:**

Interviews with key actors at the national and regional levels were analyzed using an adapted version of a conceptualization by Adam *et al.* (2019), depicting barriers to vertical policy coordination.

**Findings:**

Our results show that the main issues in the Swedish context were related to parallel sovereignty and a vagueness regarding responsibilities and mandates as well as complex governmental structures and that this was exacerbated by the unfamiliarity and uncertainty of the policy issue. We conclude that understanding the interaction between the comprehensiveness and complexity of the policy issue and the institutional context is crucial to achieving effective vertical policy coordination.

**Originality/value:**

Many studies have focused on countries’ overall pandemic responses, but in order to improve the outcome of future pandemics, it is also important to learn from more specific response measures.

## Introduction

Since healthcare in many countries is governed “at arm’s length” due to decentralization, a prominent feature of the COVID-19 pandemic responses’ around the world has been the multi-level coordination of efforts. An example was the introduction and expansion of large-scale testing for COVID-19 infection during the first year of the pandemic. *Vertical policy coordination* between different governmental and administrative levels of healthcare systems was imperative to establishing the necessary testing organization. This had to be accomplished in the midst of rapid knowledge production regarding the new disease and an exceptionally comprehensive societal crisis given its worldwide spread.

In Sweden as in other countries, an unprecedented testing organization was built up, with about 290,000 tests taken one of the last weeks of 2020 (Fredriksson and Hallberg, 2021). The overall responsibility for COVID-19 testing was, in practice, shared between national and regional authorities, resulting in an intense and complex coordination process that has been criticized for being slow and inefficient (Coronakommissionen, 2021). It is important to investigate in more detail what worked and what did not in this coordination process, and more generally, to understand the particular coordination challenges of establishing large-scale testing in different institutional contexts. This is especially true given that new and unanticipated situations, which will require rapid multi-level responses, are likely to reoccur and that decentralization can be an important asset in responding to crises such as the COVID-19 pandemic, however not without being mediated by effective coordination (OECD, 2020).

In this study, we investigate the coordination efforts related to the build-up of large-scale COVID-19 testing in Sweden during the first year of the pandemic. More specifically, the aim is to elucidate the challenges of vertical policy coordination between non-political actors at the national and regional levels regarding this policy issue. In Sweden, there was low political involvement in the handling of the pandemic and policy responses, such as large-scale testing, were largely delegated to expert bodies (Christensen and Lægreid, 2023). By focusing on barriers related to both policy-specific demands and institutional characteristics, drawing on a conceptualization by Adam *et al.* (2019), the study contributes to a better understanding of challenges in the Swedish case, as well as general knowledge about vertical policy coordination by non-political actors of a new, extensive and rapidly developing policy issue within a multi-level context.

## Background

### The COVID-19 pandemic response and the build-up of testing

The COVID-19 pandemic required various extraordinary efforts from a range of societal actors in order to control the spread of the disease and handle its consequences. In particular, governments around the world was put to the test as they needed to handle multiple new policy issues. For example, they had to decide on urgent issues such as testing, curfews and income support and business continuity packages (Boin *et al.*, 2021).

One policy issue of great importance was the build-up of large-scale COVID-19 testing (OECD, 2020). Although extensive testing was recommended by the World Health Organization (WHO) from mid-March 2020 (WHO, 2020), at the beginning of the year, it was not an evident part of a pandemic response, and there was much debate globally about the effectiveness of different approaches to large-scale testing (Mercer and Salit, 2021). The knowledge about diagnostics for COVID-19 developed rapidly during 2020, and the upscaling of testing placed a strain on the availability of necessary products (Peeling and Sia, 2023). It was in this context that organizations enabling large-scale testing were to be built up around the world, involving large numbers of actors from different organizations and at different levels of government. In Sweden this process rested on a vertical policy coordination effort between actors at the national and regional levels, owing to the decentralized structure of healthcare and infection control. In their investigation of the Swedish pandemic response, the Corona Commission was particularly critical of the disagreements between the regional and national levels on the financing of the testing policy, which they refer to as a breakdown (Coronakommissionen, 2021). With regards to the more administrative aspects of establishing large-scale testing, and the related vertical policy coordination between nonpolitical actors that we focus on in this study, their critique was subtler, while still pointing to some difficulties. For example, an unclear mandate of the regional infection control physician (Coronakommissionen, 2021, p. 743), as well as hesitancy among national and regional actors of the usefulness of large-scale testing (Coronakommissionen, 2022, p. 612) appear to have slowed down the build-up.

In light of the newfound relevance of large-scale testing in the events of pandemics, it is important to better understand the specific challenges that arose in establishing large-scale testing in Sweden and elsewhere. In addition, the build-up of testing in Sweden can serve as a case for investigating vertical policy coordination in a multi-level context during the pandemic more generally. As will be argued below, through a focus on the specificities of both the policy issue and the institutional context, this study can contribute to the broader and growing literature on the governmental responses to the pandemic.

### Literature review

The pandemic response in various countries has gained significant interest in the literature (see, e.g. Greer *et al.*, 2021). For example, the roles of politicians and experts have been discussed, and findings indicate that in some places, politicians did not heed expert advice, while in others, policy-making was largely expert-driven (Zahariadis *et al.*, 2022). Many of the studies have, to date, provided accounts of national governments’ over-arching response (see for example Askim and Bergström, 2021; Kuhlmann *et al.*, 2021). As for the response in the Nordic countries, Sweden stood out with low political involvement. Politicians relied on autonomous expert bodies for scientific advice as well as policy-making and crisis management, in particular the Public Health Agency (PHA). This agency also fronted the communication effort to citizens, contributing to the depoliticization of the pandemic response (Christensen and Lægreid, 2023). Also, in combination with high political trust, Sweden adopted a decentralized response (Zahariadis *et al.*, 2023), in contrast to a more centralized response in for example Norway (Sparf, 2022). Furthermore, Christensen *et al.* (2023) recently showed that Sweden had a more network-based response to the pandemic than the other Scandinavian countries (to which the role of experts is an important explanation). Zahariadis *et al.* (2023) has in turn showed that a managerial policy style and an autonomous bureaucracy (amongst other things) has framed the pandemic response in Sweden to a large extent.

More generally, it has been shown that the responses to the COVID-19 pandemic have been greatly influenced by the politico-administrative arrangements of specific countries (Kuhlmann *et al.*, 2021) and, for example, intertwined with the often-decentralized nature of healthcare systems. Kuhn and Morlino (2022) suggest that an essential characteristic of a nation’s capacity to manage the COVID-19 crisis has been how *decentralization* was handled through *coordination*, which, in turn, depended on the specific governing arrangements and political culture of each country. In line with this, many studies have adopted a multi-level perspective on the pandemic response and the related coordination efforts. However, a recent review suggests that “the lack of precision regarding the content of a response” is a problem (Carroll *et al.*, 2023, p. 13). Furthermore, it shows that some issues have received more attention than others and that the analysis of multi-level coordination related to specific nonpharmaceutical interventions such as testing are still rare. This despite that the character of specific policy issues has been shown to be crucial for understanding coordination during the pandemic. Many scholars, such as Boin *et al.* (2021), have for example highlighted the crisis-dimensions, such as urgency and complexity, of the COVID-19 pandemic. In particular, the implications for governments’ pandemic responses of uncertainty have been acknowledged (Boin *et al.*, 2020).

This study adds to the growing literature on the COVID-19 pandemic response by the analysis of a specific and important policy issue related to the pandemic, i.e. large-scale testing. Its contribution also lies in combining aspects of the multi-level context and the specificities of the policy issue as explanatory factors to better understand the challenges of vertical policy coordination during the pandemic. More specifically, we investigate the new and salient issue of testing and the related coordination process in Sweden. To our knowledge, few studies have examined the build-up of large-scale testing as an aspect of the governmental response to the pandemic (although the role of testing *per se* during a pandemic has been highlighted, see, e.g. Peeling and Sia, 2023). Even rarer are studies that focus on the influence of the multi-level context on the coordination efforts of non-political actors that the build-up of testing relied on. By exception, Martindale *et al.* (2021) point to some specific difficulties of this policy issue, such as tensions between levels due to its scale as well as differences in perceptions between actors regarding the correctness of testing policies.

## Theory and the Swedish case

Decentralization (along with specialization) has been an important strategy for handling public sector expansion and the increased complexity of governments. At the same time, it has accentuated the multi-level character of public systems, influencing governance and policy-making within them. The specific challenges of policy-making in decentralized contexts have, for example, been highlighted in the vast literatures on multi-level governance (for an overview, see Stephenson, 2013) and collaborative governance (Gash, 2016).

In this study, we relate primarily to the literature on *policy coordination* (Peters, 2018), and coordination is defined as “the purposeful alignment of tasks and efforts of units or actors in order to achieve a defined goal” (Lagreid and Rykkja, 2015, p. 477). What is often suggested is that the organizational structures of the public sector are a double-edged sword – a necessity as well as a hindrance – that make coordination between multiple actors imperative (Bouckaert *et al.*, 2010). A distinction is sometimes made between horizontal and vertical coordination, where the latter has received increased attention recently (Adam *et al.*, 2019). The vertical aspect is not least seen as an essential aspect regarding healthcare systems, where decentralized structures are handled by different coordination mechanisms that “provide linkages between the different subsystems” (Vrangbæk, 2007, p. 57). An example of such a coordination mechanism is networks (O’Flynn, 2013) and, more specifically, networks consisting of non-political actors (Behnke, 2019). While the over-arching frames for policy initiatives are stipulated by the political level, such networks hold an important role in the actual coordination and implementation of policy in multi-level contexts such as Sweden.

When it comes to understanding successful coordination processes or outcomes, for example through networks, there are different perspectives available. O’Flynn (2013) lists a number of enablers as well as barriers, and for her and other authors, *formal structures* is one factor that reoccurs. In line with this, a distinction can be made between a structural and a cultural perspective, where the former pays attention to how the structures of public administration influences decision-making and coordination (Lagreid and Rykkja, 2015). In this study, we similarly concentrate on what Adam *et al.* (2019, p. 501) refers to as political-administrative structures and in particular those structures originating from multiple governmental levels and decentralization. We emphasize this structural aspect because it has been demonstrated to be important for our understanding of pandemic responses (Kuhn and Morlino, 2022).

In addition, another factor influencing coordination efforts through networks in the public sector is the character of the policy issue. It has been suggested that contemporary issues, such as antimicrobial resistance and climate change, are increasingly complex, and some have for example been characterized as “creeping”, “wicked” and so forth (Boin *et al.*, 2020; O’Flynn, 2013). Organizational structures, for example those related to multiple jurisdictional levels, may be insufficient for handling complex issues and the “problem structure” matter for coordination (Lagreid and Rykkja, 2015). Also, engaging in a policy issue for the first time, or in one characterized by uncertainty, can influence related vertical coordination, making it more difficult to achieve (Adam *et al.*, 2019). In line with this, a combined focus on the policy issue and multi-level governance has been suggested (Paquet and Schertzer, 2020).

In the following, we will analyze this combination in the case of large-scale testing in the multi-level context of Sweden. First, we give an overview of testing as a policy issue in the Swedish context more generally.

### Large-scale testing in relation to the Swedish administrative model

The first case of COVID-19 in Sweden was discovered on January 31 2020 and it was declared a disease dangerous to society on February 1, entailing that testing and contact tracing – if deemed necessary by a physician – became mandatory by law. The risk for community transmission was deemed very high on March 10. In connection to this Sweden abandoned its initial test and trace strategy (Saunes *et al.*, 2021) and focused instead on testing priority groups (see Table 1). On March 31, the national government assigned the PHA the task of rapidly increasing the capacity for COVID-19 testing (Socialdepartementet, 2020a) and on June 4 the task of ensuring the possibility of large-scale testing and the resumption of contact tracing (Socialdepartementet, 2020b). In this article, we focus on the policy of large-scale testing represented by the two government assignments, focusing primarily on the upscaling of capacity for *taking* and *analysis* of tests (but not tracing). For a more detailed description of the build-up of testing in Sweden, see Fredriksson and Hallberg (2021).

During 2020, there was substantial debate in Sweden about the role of decentralization and the Swedish administrative model in slowing the build-up of large-scale testing (Fredriksson and Hallberg, 2021). With 21 regions (and in part the 290 municipalities) responsible for its funding and provision, the healthcare system in Sweden is considered highly decentralized (OECD, 2020). The two local levels, the regions and the municipalities, have far-reaching responsibilities and their autonomy is laid down in the constitution. Furthermore, they are governed by locally elected politicians. At the same time, Sweden is a unitary state, meaning for example that the exact division of tasks between the national, regional and local levels can be altered by central government (Statskontoret, 2020), and has consequently been classified as a “decentralized unitary state” (Feltenius, 2015).

The Swedish administrative model also entails a dualism at the national level; government agencies hold a comparatively independent position vis-à-vis the government. Due to a principle of responsibility in times of crisis, the administrative model remains the same as during ordinary circumstances (Statskontoret, 2020). Taken together, this means that healthcare governance in Sweden, both during normal circumstances and in times of crisis, is characterized by “soft” steering through information as well as coordination through informal contacts and agreements (Maino *et al.*, 2007; Pierre, 2020), not least between civil servants and experts (Saunes *et al.*, 2021; Zahariadis *et al.*, 2023). In this, the Swedish Association of Local Authorities and Regions (SALAR) holds an important position by representing the regions in negotiations with the national government (Statskontoret, 2020).

Also during the COVID-19 pandemic, central decision-making authority in Sweden was to a comparatively large extent delegated to government agencies (Askim and Bergström, 2021). In particular, the PHA had a strong position. In line with its role as an expert agency, the PHA continuously published guidelines, the most important being the recommendation for whom to test for COVID-19, which until June 17 2020 specified four priority groups, see Table 1. Enjoying a high administrative policy capacity due to autonomy, staffing and aspirations (Zahariadis *et al.*, 2022), the PHA was in practice delegated extensive policy responsibility (Christensen and Lægreid, 2023).

Other important actors include the Swedish Civil Contingency Agency (MSB) responsible for issues related to public safety and emergency management, the County Administrative Board (CAB) which are the national government’s representatives at the regional level and SALAR. Furthermore, the infection control organization is delegated to the regions. A lead infection control physician is appointed by each region and during the pandemic they worked closely with healthcare professionals responsible for the build-up of regional testing units. Importantly however, the decentralization of infection control is not as far-reaching as that found for example in Norway (Askim and Bergström, 2021). According to the specific law regulating communicable disease control (2004, p. 168), the PHA is responsible for coordinating communicable disease control at the national level, while the regions are responsible for taking necessary measures in their area. Regarding testing, there is also in practice a separation between *taking tests* and *analyzing tests*, with the latter being more of a shared task for national and regional actors.

As described above, the build-up of testing in this context was a complex and difficult process (Coronakommissionen, 2021). In the following, we will extend on these challenges by analyzing the implications for vertical policy coordination related to large-scale testing in Sweden of both policy-related and institutional barriers. This will be achieved by using an adapted analytical framework, presented in more detail below.

## Analytical framework

In order to account for both structural and policy-specific features in our analysis, we employ a conceptualization by Adam *et al.* presented in 2019 consisting of nine potential determinants of successful vertical policy coordination. More specifically, Adam *et al.* include three factors related to policy-specific demands, institutional and political barriers, respectively (see Table 2).

Given this study’s focus on coordination between non-political actors, and our specific interest in the interaction between the policy issue and structural aspects, the political dimension in the Adam *et al.* model was excluded. As described above, the administrative processes of vertical coordination related to the build-up of large-scale testing were to some extent detached from the more over-arching political process, and for the involved civil servants, the political aspects suggested by Adam *et al.* (for example party competition and regional parties) were less relevant. At the same time, the conceptualization of Adam *et al.* was found useful for other reasons. Importantly, they include a number of factors related to the specific policy issue (“policy-specific demands”) since its complexity is thought to impact the incentives for reaching out and performing coordination. Furthermore, they focus on vertical coordination specifically (shown by the specific category of parallel sovereignty), which is in line with this study’s focus on coordination between different government levels. Therefore, we formulate an analytical framework based on the six categories related to policy-specific demands and institutional barriers (described in more detail below). It should be noted that this does not entirely leave out the role of the national government from the analysis, but that this is captured by the category of governmental structures. Related to this is another specification related to what is pointed out by Behnke (2019), namely that it is not always useful to see central government as monolithic. In the Swedish case, due to the dualism at the national level, this is arguably an important distinction and the code national level was therefore included in the operationalization of the category Governmental structures (see Table 3).

The study’s framework, based on the descriptions by Adam *et al.* (2019), with its adaptations, codes and operationalizations are presented in Table 3.

Regarding the policy-specific demands, *frequency* suggests that the number of times a policy issue has been coordinated is a factor with the potential to influence vertical policy coordination. This relates to the knowledge and understanding the involved actors have of the issue and the different policy instruments at hand, as well as the policy’s legitimacy among implementers. *Specificity* suggests that the success of vertical policy coordination can be influenced by the homogeneity/heterogeneity of the target group of a policy and that it becomes more difficult if a “one size fits all” approach is hard to achieve. *Uncertainty* suggests that the level of uncertainty regarding the extent of a policy issue and the effectiveness of a policy design affects vertical policy coordination.

Regarding institutional barriers, *coordination venues* suggests that vertical policy coordination is facilitated if there are institutionalized arenas for coordination and also if *de facto* rather than *de jure* implementers participate. *Parallel sovereignty* suggests that the extent to which a policy issue is under “competing jurisdictions” may affect vertical policy coordination. *Governmental structures* suggest that complexity because of, for example, multiple implementing actors (interpreted here as the 21 regions), can be a barrier to vertical policy coordination.

## Methods and analysis

In this case study, we interviewed key actors of the organizations involved in the build-up of COVID-19 testing in Sweden during 2020. We focused on civil servants at the national and regional levels closely involved in the related coordination process. More specifically, all respondents were either in charge of or actively engaged in decision-making regarding the build-up of testing, giving them unique insight. Together they represent the most important stakeholders involved in the process of establishing a large-scale testing apparatus (see Table 4). As described in the Background section, the regional level was by far the most important local actor in the build-up of large-scale testing, which is why no municipal actors were included.

Potential respondents were purposefully identified using a snowball technique and then informed and asked to participate in the study via e-mail. Written informed consent was obtained from all participants. In total, 13 respondents were interviewed, see Table 4.

As key actors were recruited in the midst of an intense phase of the pandemic, the research team utilized their networks in certain regions to encourage potential respondents to take the time and prioritize participating in the study. This resulted in actors from three regions being included, in addition to key actors at the national level. The regions had similar crisis trajectories (i.e. relatively high infection rates during the spring of 2020 compared to other parts of Sweden) and thus the build-up of testing was equally urgent. At the same time, the three regions have different population size and structural and demographic differences, aiming to provide a fuller account of challenges associated with vertical policy coordination during the initial phase of the pandemic.

A method based on interviews was used in order to gain a deep understanding of the processes and perspectives of the different actors regarding the build-up of the testing. To ensure the accuracy of the respondents’ accounts and strengthen the validity of the study, the interviews took place during the spring of 2021, in close connection to the actual coordination process. The interview guide (see appendix) was not based on the analytical framework, but aimed to capture a detailed chronological description of the build-up of testing (for the four specific phases used, see Fredriksson and Hallberg, 2021). Structuring the interviews in this way was deemed important given the complexity of the process. AH conducted all interviews (11 of them jointly with MF). All interviews were conducted and recorded via video conference (Zoom) and then transcribed verbatim. The study protocol was approved by the Swedish Authority for Ethical Review (no. 2020–05732).

The accounts of the coordination process by different actors were analyzed using the adapted framework (described in detail above) based on Adam *et al.* (2019). The coding of the accounts was “neutral” in the sense that everything that the respondents stated that related to a category, both negative and positive, was coded (also counteracting selectivity in the use of data). In practice, the analysis consisted of marking up the interview transcripts using color coding and comments in Microsoft Word. In a next step, all codes for each category were gathered in a separate document, as a first condensation of the material. This allowed for the authors to gain an overview of all the respondents’ accounts and of differences and similarities among them. The most frequent and important themes of each category were then extracted. In this process, analytical rigor was ensured through the last author (MF) discussing and verifying the initial coding by the first author (AH). None of the authors involved in the analytical process hold a position that entails a risk of bias or undue influence on the research.

## Results

In the following, the specific expressions of the barriers are presented and analyzed with regards to how they affected vertical policy coordination.

### Policy-specific demands

#### Frequency

All respondents expressed that large-scale testing during a pandemic was a new area of coordination. For example, a region representative said:

[…] there were no plans for that in Sweden, that we were to test everyone with symptoms, that is something completely new […] (interview 5: region 3)

The novelty consisted both of the *idea* of large-scale testing as a nonpharmaceutical response during a pandemic and the *scale* of the operation. Regarding the latter, several of the region representatives bore witness to how the expectations by national actors regarding the capacity to quickly scale up testing did not match the novelty and complexity of the task (although the difficulties in meeting the expectations varied between the three regions, see also Governmental structures):

[…] once it was decided that we were to have large-scale testing, they relied on the regions to manage it, but there was no chance really. We are not used to opening testing stations around town and the logistics were not set up quickly and efficiently […] (interview 4: region 2)

An unfamiliarity with the issue was also reflected throughout the coordination process in that the national actors described that they had limited experience of their new tasks, for example for the PHA to provide the regions with additional laboratory capacity through new procurements. Another example was the government assigning MSB to develop a tool to support the regions in identifying individuals belonging to priority group 3 (i.e. individuals with important functions in society) which was described as a new task that did not work out entirely as planned (*interview 10*).

At the same time, there was a lack of animosity among the national and regional actors involved, except regarding the expectations on capacity. Rather, there was a common understanding of the novelty of the issue and of the need to find solutions to a number of areas related to large-scale testing such as potential displacement effects and patient integrity and safety. The importance of safeguarding trust among the implementers in the regions while expanding testing was expressed by the PHA *(interview 8)*.

As mentioned, another aspect of frequency was that large-scale testing was a new *idea.* As this is also connected to the state of knowledge regarding testing it will be discussed further under the heading *Uncertainty.*

#### Specificity

The PHA’s testing recommendations can be seen as temporary specification of the target group for large-scale COVID-19 testing. While there was consensus among national and regional actors regarding the definition and handling of the first two prioritized groups (i.e. patients at hospital wards and health and social care staff) which facilitated vertical policy coordination, greater problems related to specificity were encountered later on concerning priority group 3. The representative of SALAR described difficulties in defining who belonged to this group, something that was also confirmed by region representatives:

There was almost no one who did not think they had a function of importance to society […] So the priority groups were, well … It took a lot of energy to try to interpret and sift through it […] (interview 2: region 1)

With the expansion of testing to all individuals with symptoms (priority group 4) in June, new aspects of heterogeneity in the target group arose and the regions chose different ways of organizing the testing in order to make it available to different population groups. A representative of the PHA further described the heterogeneity related to large-scale testing for COVID-19 in relation to intensive care:

[…] one might think that dealing with a patient in intensive care is of course so much more complex, but then there is […] a more limited crowd […] (interview 8)

#### Uncertainty

Several respondents described uncertainty, especially regarding the effectiveness of large-scale testing in slowing down the spread of COVID-19. Related to this, respondents from the regions bore witness to the PHA communicating early on that extensive testing was not appropriate after societal spread had been detected:

It was explained to us that we now have community transmission, we cannot prevent the infection from gaining ground in Sweden, now this extensive testing […] and contact tracing no longer has a place […] (interview 1: region 1)

The PHA on their part described that they related to the current state of knowledge, for example when it came to making changes in the testing recommendations, and that they sometimes had to “wait for some knowledge” *(interview 9).*

Especially in Region 1, a strong resistance towards large-scale testing before the second wave of infection in the fall of 2020 was described, related to this uncertainty regarding effectiveness. According to one respondent, the laboratory staff thought that it was “crazy” to test extensively when the spread was relatively low *(interview 2: region 1)*. The PHA also bore witness to this ambivalence within some regions:

[…] there was a clear ambivalence in the regions regarding the value of establishing large-scale testing because no other pandemic has been dealt with in that way. (interview 8)

### Institutional barriers

#### Coordination venues

While there were institutionalized forums for coordination from the start, for example in the form of weekly meetings between the PHA and all the infection control physicians together, the respondents’ accounts showed in different ways that theses venues were insufficient. For example, it was described that the PHA initially communicated the testing recommendations to the infection control physician, but not directly to the de facto implementers in the regions, i.e. those responsible for the regions’ testing units.

The initial coordination venues were however extended during the spring and further formalized thereafter. For example, one-to-one meetings between the PHA and the regions, sometimes including both infection control and the testing units, were established, showing an adaptability of the existing coordination venues. One of the PHA representatives described how it affected coordination to have both types of region representatives (infection control and testing) in the same meeting, namely that it “sped up the whole thing” *(interview 9)*.

Even with the enhanced communication between the PHA and the regions, SALAR constituted an important and “obvious” actor for the PHA to interact with in order to get insight into the internal operations of the regions according to a CAB representative *(interview 13)*, referring to the communication among national actors in a network established in June. The PHA described this as SALAR being more involved in the “basic operations” in the regions than themselves *(interview 8)*.

#### Parallel sovereignty

Much ambiguity regarding the responsibility for testing was found in the respondents’ accounts. For example, the PHA stated that their point of departure was the principle of responsibility and that this was something they repeatedly had to explain. Furthermore, the PHA expressed clearly that their view was that the regions themselves had to decide on the volume of testing in relation to the region’s needs, indicating that they saw their recommendations for testing only as guidance for prioritization. At the same time, in all the interviews with region representatives, it was stated that the testing recommendations issued by the PHA were followed strictly and limited the extent of testing. There was however some variation in the interpretation of the recommendations among the regions included in this study. Some described the document more clearly as *restricting* testing while others saw it as a guideline for *prioritization,* which however became quite directive in combination with the lack of capacity.

The interviews contain several more expressions of an ambivalence about the mandate to make decisions regarding testing, for example by one region representative:

Should you sort of step away from that and say no, so, in our region, we test all those with symptoms of COVID as well. Of course you could do that, no one would stop you, but it would be a huge deviation from the national recommendation really. (interview 1: region 1)

Although there was seemingly an intention by the PHA to keep the operative responsibility for testing with the regions according to the principle of responsibility, their support to the regions was eventually reinforced by a government assignment regarding procurement of additional laboratory capacity. The increased support, which can be seen as a shift in the division of responsibility between the national and regional level, was described by one of the PHA representatives as being a result of the slow scale-up in some regions:

[…] we have always had a dialogue with the Government Offices about the possibilities and we saw that it was slow and sluggish and that there was a need to give the regions more operational support and were sort of positive about it, even though it was a completely new role for us. (interview 8)

In line with this, it was stated by a region representative that the additional laboratory capacity from the PHA made it much easier to “press the button” and test everyone that wanted a test *(interview 3: region 2)*.

#### Governmental structures

The multitude of implementing actors, i.e. the 21 regions, was at several times referred to as a complicating factor, for example when it came to purchasing goods and IT support. One example of how this affected the coordination process was the high workload for national actors communicating separately with 21 different actors (which eventually became the way of communicating, see Coordination venues):

[…] it has not made it easier that there are twenty-one different [regions] … and there have really been twenty-one different ways of doing it. So that it has meant more work, more meetings […] (interview 9)

Apart from the sheer number of actors to coordinate, this quote also indicates regional variation on a number of aspects. For example, the practical problems related to the establishment of testing organizations varied in the three regions in our study, ranging from difficulties of organizing the *taking of tests* to issues with ensuring of the *analysis of tests*. As a result, the PHA had to offer different logistic solutions, for example a number of alternative testing flows and extra capacity adapted to the different regions, depending on the nature of the region’s problems. Another variation among the regions acknowledged by some respondents were differences in capacity for establishing large-scale testing. There appears to have been an advantage for larger regions (although a representative of the smaller region also acknowledged the benefits of being a smaller region when it came to getting an overview and knowing each other). While some of the variation found exemplifies how the build-up of testing was adapted to local conditions, it appears as if large-scale testing and not least the novelty of the policy issue (see Frequency) resulted in an unusual and sometimes unwarranted variation of solutions.

It was further expressed by both of the PHA representatives that testing constituted a complex chain that is dependent on several special purpose authorities at the regional level. Most prominent were the laboratories, which were essential in analyzing the tests taken by the regional testing units. In the interviews with representatives from regions 1 and 3, it was indicated that their laboratories were occasionally out of the loop and did not receive critical information. Also, in Region 1, it was stated that their main laboratory had its own view on the extent and effectiveness of testing (see also Uncertainty). Besides these potential problems related to communication with the laboratories, the overall view provided by the respondents was that of well-functioning cooperation at the regional level. All but one of the region representatives acknowledged this specifically, for example in terms of “teamwork” *(interview 1: region 1)* or “very close cooperation” *(interview 3: region 2)*.

Lastly, the separation at the national level between the PHA and the government seems to have played a role in the efforts to achieve vertical policy coordination. For example, in May the national government presented a particular number describing Sweden’s analysis capacity – 100,000 tests per week – which by some was interpreted as a target (in Region 2 they tried to reach their share of it based on the region’s population size). Several of the region representatives as well as one of the CAB representatives *(interview 11)* expressed that they perceived the number as discrepant in relation to the PHA’s recommendations for testing. One of the PHA representatives described that the number was not intended as a target, at the same time stating that “it never hurts to have something to measure up against” *(interview 8)*.

## Discussion

The aim of this study was to elucidate challenges of vertical policy coordination between non-political actors during the build-up of large-scale COVID-19 testing. In the following section, we summarize and discuss the most important findings from interviews with key stakeholders, both for Sweden and more broadly.

Firstly, as a result of different views on the mandate to decide on the extent of testing (exemplified by the Swedish regions’ varying and generally cautious interpretations of the PHA’s testing recommendations), regional actors appear to have sometimes awaited national actors’ actions and vice versa. The continuous expansion and unexpected scale of COVID-19 testing, in combination with a shared responsibility, appear to have complicated vertical policy coordination. More generally, this suggests a combined influence of the policy issue itself and the multi-level context on coordination through a dynamic similar to what Boin and t Hart (2014) refer to as an upscaling conflict likely to occur in times of crisis.

For Sweden, it has already been acknowledged elsewhere that the role of the infection control physicians needs to be clarified in this regard (Coronakommissionen, 2021) and our findings are in line with this. Also related to *parallel sovereignty* in Sweden specifically is the ambiguity regarding the role of the PHA, interpreted as more of a policy-maker by the region representatives and merely as a provider of knowledge support by the PHA themselves. This ambiguity is potentially connected to the PHA’s strong position in the overall national pandemic response (where they, e.g. led daily press conferences) and an indistinct leadership by the national government (Coronakommissionen, 2022). Sweden relied more heavily on professional expertise (e.g. scenario modeling, contingency planning and mobilizing response capacity) than political expertise (Boin *et al.*, 2021). Also, for comparison, testing proved to be a more complicated issue in terms of *shared responsibility* than for example intensive care, where the National Board of Health and Welfare appears to more clearly have had a supporting role when the regions had to scale-up capacity (and the regions could circumvent the government agency when needed, see Coronakommissionen, 2021, p. 464).

Intensive care was also a less *contested* part of the pandemic response than large-scale testing of the population. While it is not possible from our material to explain precisely how this greater *uncertainty* regarding the appropriateness of large-scale testing made vertical policy coordination difficult, it is likely that the sometimes strongly differing opinions among actors at the regional level was a complicating factor in Sweden. Our results suggest that the PHA was initially hesitant to large-scale testing (although the decision to focus testing on vulnerable groups and abandon contact tracing was also a result of a lack of capacity in the regions). This is in line with the agency’s reliance on pre-existing rather than new evidence (Olofsson *et al.*, 2022) and a clear focus on evidence-based knowledge that has been criticized (Christensen and Lægreid, 2023). In light of the strong influence of the PHA when it came to the testing recommendation (as shown above), it is possible that the hesitancy regarding the effectiveness of large-scale testing had a similar effect, resulting in a lingering uncertainty and differing opinions in the regions. Of particular interest in this regard is the finding that the laboratories were sometimes “out of the loop”. Apart from the importance of potential variation between regions in this regard in the Swedish case, it also shows how a combined influence of governmental structures, such as multiple implementing actors, and uncertainty complicated the common coordination effort. This potential interaction between structural factors and the characteristic of the policy issue should be considered relevant for other new and evolving policy issues as well.

Another finding related to *governmental structures* is the substantial variation among the implementing actors, enhanced in the case of COVID-19 testing by the unfamiliarity with the issue. In Sweden, this caused great diversity of testing organization set-ups and challenges in the regions as well as difficulties of getting a national overview. The number and size of the regions is a longstanding discussion in Sweden (Ansvarskommittén, 2007; Indelningskommittén, 2018), and our results show that the current division was not optimal for vertical policy coordination of this new and complex issue. Our study further shows that there were some practical issues related to the unfamiliarity of vertical policy coordination on this scale, for example the accreditation and involvement of new laboratories. These problems related to *frequency* appear however to have been mediated by a common understanding among the involved actors of the issue at hand, indicating that features of a “bureaucratic network” between the regional and national levels (Behnke, 2019) may have facilitated vertical policy coordination. In line with this, there were institutionalized *coordination venues* available from the start in Sweden, and these were also adapted continuously. Thus, coordination venues did not constitute a major barrier to vertical policy coordination in the long run, although there were initially demarcations and gaps that obstructed communication between national and regional actors. Clearly, the complexity of testing required coordination forums involving more actors than were initially available. Again to compare, a similar tendency of adaptability and flexibility could be seen in the scale-up of intensive care capacity (Coronakommissionen, 2021).

Lastly, when it comes to *specificity*, the heterogeneity in the target group does not appear to have constituted a substantial barrier to vertical policy coordination, with the exception of the difficulties of defining who belonged to priority group 3. A possible explanation is that the heterogeneity in this case was mainly related to the *collection of tests*, which is more clearly a responsibility of regional actors alone. Regarding priority group 3 however, our respondents described disputes and unforeseen difficulties. This suggests that the benefits of increased specificity for vertical policy coordination, as proposed by Adam *et al.* (2019), are not guaranteed but dependent on the ability to define a certain subgroup and how the specification relates to the division of responsibility.

To sum up, the Swedish case demonstrates how institutional and policy-specific barriers, and their interaction, influenced coordination related to the build-up of large-scale testing. Among other things, complexity and uncertainty (causing differing opinions among the involved actors) along with fragmented governmental structures, was a complicating factor. This was mediated however by the ability to adapt the forums for coordination and a common understanding among the actors of the boundary-spanning network (suggestively related to the unusually expert-based and professionalized response in Sweden). Our results also showed a general vagueness regarding roles and differing views among regional and national actors on mandates. Importantly, using the terminology of Adam *et al.*, this vagueness was accentuated by policy-specific characteristics such as frequency, and this combination was difficult to handle. Furthermore, the novelty of large-scale testing in combination with many implementing actors (the 21 regions) complicated vertical policy coordination.

## Limitations

This study has some limitations. First, although our respondents hold key positions in the build-up of the testing organization in Sweden, they constitute a limited number of the involved actors. In particular, it is a limitation that no representatives from the regional laboratories were included. Second, we only included 3 of 21 Swedish regions in this study which limits the possibility to draw general conclusions about the vertical policy coordination within Sweden. We do, however, believe that our main findings are relevant and transferable to Sweden as well as other similar policy contexts. Lastly, the chosen framework restricts the analysis to a rather institutional perspective, and further studies focusing more on the influence and agency of individuals could significantly contribute to our understanding. Neither does our study acknowledge for example norms or culture as potential explanatory factors to any great extent. While we believe that the framework provides a relevant perspective in this particular case, we encourage taking other perspectives into account when evaluating this complex process. Related to this complexity, it is important to note that the comprehensiveness of the issue of large-scale testing for COVID-19 has of course caused restraints regarding resources that have also had implications for the build-up of testing. Also, to our knowledge it is unclear to what extent the testing organization in Sweden was more accurate and safer overall compared to other countries, and the speed of the scale-up of a testing organization should be weighed against this.

## Conclusion

Unsurprisingly, the build-up of large-scale testing in a multi-level system, such as Sweden, posed several challenges for vertical policy coordination. The contribution of our study is a deeper understanding of the encountered issues. We achieved this by investigating barriers related to both policy issue characteristics and the institutional context, exploring how these two aspects interact.

For Sweden specifically, our study confirms the findings of the Swedish Corona Commission about problems related to the shared responsibility and uncertainty of the policy issue. In sum, while some things, such as the cooperation at the regional level and the common understanding of practical matters between regional and national actors worked well, there is a recognized need in the Swedish context to develop strategies to better handle the implications of parallel sovereignty and governmental structures for vertical policy coordination of comprehensive policy issues. This could include clarifying mandates and making it easier to solve sudden issues of unclear responsibilities during a crisis, as well as identifying what aspects of large-scale testing that would gain from a more national approach (in order to decrease unwarranted complexity and diversity). With its unusually depoliticized response to the pandemic, consideration must also be given to the potentially quicker upscaling of testing in countries where political influences played a more prominent role during the pandemic, such as Norway and Denmark. However, this should be weighed against the benefits of an expert-based network for handling such a highly technical issue. It would also be of interest to further investigate how the handling of COVID-19 testing affected outcomes such as infections and deaths during the pandemic, which were considerably lower in neighboring countries (Christensen and Lægreid, 2023).

While Sweden represents a specific multi-level context with specific dynamics, the findings regarding a policy issue common to most countries worldwide should have broader relevance. Particularly, it should give insights about interactions between the policy issue of large-scale testing and the institutional context of other multi-level systems. Given the newfound relevance of large-scale testing for handling pandemics, the results of this study can ideally inform and help us in preparing for similar situations in the future.

## Figures and Tables

**Table 1:** The priority groups presented in the first national strategy for COVID-19 testing

Group	Description
1	Patients falling ill with acute infections in need of inpatient care, inpatients at hospitals, individuals belonging to any risk group and residents in care and in institutions
2	Healthcare and social care staff
3	Individuals having other functions of importance for society
4	Other relevant parts of society

**Source(s):** Public Health Agency of Sweden (2020)

**Table 2:** Conceptualization of barriers to vertical policy coordination

Policy-specific demands	Institutional barriers	Political barriers
Frequency	Coordination venues	Party competition
Specificity	Parallel sovereignty	Regionalist parties
Uncertainty	Governmental structures	Political pressure to act

**Source(s):** Adam *et al.* (2019)

**Table 3:** Analytical framework and operationalizations

Category	Code and operationalization
Frequency	*Frequency: general.* Reference to degree of experience of coordination *Frequency: implementation structures.* Descriptions of information/knowledge about methods/policy instruments for coordination
	*Frequency: learning.* Descriptions of increasing experience of coordination; Description of increasing understanding/knowledge about methods/policy instruments for coordination *Frequency: legitimacy.* Description of support or distrust towards coordination attempts
Specificity	*Specificity: heterogeneous priority.* Descriptions of heterogeneity resulting from the priority groups and reference to its effect on coordination *Specificity: heterogeneous general.* Descriptions of other types of heterogeneity in the target group and reference to its effect on coordination
Uncertainty	*Uncertainty: extent.* Descriptions of certainty/uncertainty regarding the extent of infection spread *Uncertainty: effectiveness.* Descriptions of certainty/uncertainty regarding the effectiveness of testing for mitigating infection
Coordination venues	*Coordination venues: general.* Descriptions of institutionalized forums for coordination *Coordination venues: de facto/jure.* Descriptions of de jure/de facto participation in forums for vertical coordination; Reference to the effect on coordination of de jure/de facto participation in the forums for coordination
Parallel sovereignty	*Parallel sovereignty: competing jurisdictions/separated spheres.* Perceptions/descriptions regarding who is responsible and regarding self-government more generally; Perceptions/descriptions regarding the division of responsibility for the specific policy issue
Governmental structures	*Governmental structures: special purpose authorities.* Descriptions of multiple actors responsible for specific tasks related to testing at the regional level; Descriptions of effects on coordination of these regional structures *Governmental structures: cooperation at the regional level.* Descriptions of cooperation at the regional level
	*Governmental structures: regional variation.* Descriptions of multiple regional implementing actors; Descriptions of differences between regional implementing actors; Descriptions of effects on coordination of multiple/differing regional implementing actors *Governmental structures: national level.* Descriptions of multiple actors at the national level and the effects of this on coordination

**Source(s):** Modified by the authors from Adam *et al.* (2019)

**Table 4:** The respondents

Regional level	National level	County administrative board
Region 1 infection control (interview 1)	SALAR (interview 7)	CAB 1 (interview 11)
Region 1 testing (interview 2)	PHA1 (interview 8)	CAB 2 (interview 12)
Region 2 infection control (interview 3)	PHA2 (interview 9)	CAB 3 (interview 13)
Region 2 testing (interview 4)	MSB (interview 10)	
Region 3 infection control (interview 5)		
Region 3 testing (interview 6)		

**Source(s):** Authors’ own creation

## Appendix Interview guide

### Phase 1 (Jan-Feb 2020)

*Example questions national actors*

1. How did your organization prepare for/support the establishment of testing in the regions?
2. What form did the communication between the Public Health Agency and the microbiological laboratories take during January and February?

*Example questions regional actors*

1. Can you describe how you worked to establish testing for COVID-19 in your region during this period? What kind of support did you have from national actors in this work?
2. To what extent did you discuss the division of responsibility between regional and national actors during this period?

### Phase 2 (March-April 2020)

*Example questions national actors*

1. What was the rationale for focusing testing on the priority groups in March 2020? To what extent was this decision discussed with regional actors?
2. Was there an awareness of a potential for confusion with regards to the responsibility for priority group 3 and 4 at this point?

*Example questions regional actors*

1. How and when did you start building up testing capacity for COVID-19 in your region?
  - In what way did the Public Health Agency have insight in this work?
  - What kind of communication did you have with SALAR during this period?
2. What did your work with building capacity for analysis specifically consist of?
  - What information did you get from the PHA about this part of the testing organization?
3. How did cooperation between the regional infection control and those responsible for establishing the testing organization work in your region?

### Phase 3 (May-June)

*Example questions national actors*

1. In what way were you [the PHA or SALAR] involved in the policy discussions around transitioning to large-scale testing?

Example questions regional actors

1. In your view, what was the reason for the differences in regional capacity for *testing* and national capacity for *analysis* during May 2020? How and what did your region communicate to the PHA/SALAR during this period?

### Phase 4 (July-Nov)

*Example questions national actors*

1. How did the coordination of testing continue during the summer and the beginning of the fall of 2020?
  - How did you communicate with the regions regarding a possible “second wave” and the continuous need for testing?
2. Finally, what is your general view on how the coordination nationally and vis-à-vis the regions has worked?

*Example questions regional actors*

1. How did the work regarding large-scale testing continue in your region during the summer and the beginning of the fall of 2020?
  - Did the support you received from the PHA and the government change during this period?
2. Finally, how was the support and coordination from the national level experienced in your region?

Source(s): Authors’ own creation

## References

[ref001] Adam, C., Hurka, S., Knill, C., Peters, B.G. and Steinebach, Y. (2019), “Introducing vertical policy coordination to comparative policy analysis: the missing link between policy production and implementation”, Journal of Comparative Policy Analysis: Research and Practice, Vol. 21 No. 5, pp. 499-517, doi: 10.1080/13876988.2019.1599161.

[ref003] Ansvarskommittén (2007), Hållbar Samhällsorganisation Med Utvecklingskraft (SOU 2007:10), Stockholm, available at: https://www.regeringen.se/rattsliga-dokument/statens-offentliga-utredningar/2007/02/sou-200710/

[ref004] Askim, J. and Bergström, T. (2021), “Between lockdown and calm down. Comparing the COVID-19 responses of Norway and Sweden”, Local Government Studies, Vol. 48 No. 2, pp. 291-311, doi: 10.1080/03003930.2021.1964477.

[ref005] Behnke, N. (2019), “How bureaucratic networks make intergovernmental relations work: a mechanism perspective”, in Behnke, N., Broschek, J. and Sonnicksen, J. (Eds), Configurations, Dynamics and Mechanisms of Multilevel Governance, Palgrave Macmillan, pp. 41-59.

[ref006] Boin, A. and t Hart, P. (2014), “Aligning executive action in times of adversity”, in Lodge, M. and Wegrich, K. (Eds), Executive Politics in Times of Crisis, Palgrave Macmillan, London, pp. 179-196.

[ref007] Boin, A., Lodge, M. and Luesink, M. (2020), “Learning from the COVID-19 crisis: an initial analysis of national responses”, Policy Design and Practice, Vol. 3 No. 3, pp. 189-204, doi: 10.1080/25741292.2020.1823670.

[ref008] Boin, A., Mcconnell, A. and t Hart, P. (2021), Governing The Pandemic: the Politics of Navigating a Mega-Crisis, Palgrave. doi: 10.1007/978-3-030-72680-5.

[ref009] Bouckaert, G., Peters, B.G. and Verhoest, K. (2010), The Coordination of Public Sector Organizations, the Coordination of Public Sector Organizations, Palgrave Macmillan. doi: 10.1057/9780230275256.

[ref010] Carroll, B.J., Brummel, L., Toshkov, D. and Yesilkagit, K. (2023), “Multilevel governance and responses to the COVID-19 pandemic: a systematic literature review”, Regional and Federal Studies, pp. 1-22, doi: 10.1080/13597566.2023.2292997.

[ref011] Christensen, T. and Lægreid, P. (2023), “Crisis management of the COVID-19 pandemic: the nordic way and Swedish exceptionalism”, in Cheung, A.B.L. and Van Thiel, S. (Eds), Crisis Leadership and Public Governance during the Covid-19 Pandemic: International Comparisons, World Scientific Publishing, pp. 289-323.

[ref012] Christensen, T., Jensen, M.D., Kluth, M., Kristinsson, G.H., Lynggaard, K., Lægreid, P., Niemikari, R., Pierre, J., Raunio, T. and Adolf Skúlason, G. (2023), “The Nordic governments' responses to the Covid-19 pandemic: a comparative study of variation in governance arrangements and regulatory instruments”, Regulation and Governance, Vol. 17 No. 3, pp. 658-676, doi: 10.1111/rego.12497. PMC9538262 36246344

[ref013] Coronakommissionen (2021), Sverige Under Pandemin (SOU 2021:89), Stockholm, available at: https://www.regeringen.se/rattsliga-dokument/statens-offentliga-utredningar/2021/10/sou-202189/

[ref014] Coronakommissionen (2022), Sverige Under Pandemin (SOU 2022:10), Stockholm, available at: https://www.regeringen.se/rattsliga-dokument/statens-offentliga-utredningar/2022/02/sou-202210/

[ref015] Feltenius, D. (2015), “Subnational government in a multilevel perspective”, in Pierre, J. (Ed.), The Oxford Handbook of Swedish Politics, Oxford University Press.

[ref002] Fredriksson, M. and Hallberg, A. (2021), “COVID-19 testing in Sweden during 2020 – split responsibilities and multi-level challenges”, Frontiers in Public Health, Vol. 9, doi: 10.3389/fpubh.2021.754861. PMC8639858 34869171

[ref016] Gash, A. (2016), “Collaborative governance”, in Ansell, C. and Torfing, J. (Eds), Handbook on Theories of Governance, Edward Elgar Publishing, pp. 497-509.

[ref017] Greer, S.L., King, E.J. and Massard da Fonseca, E. (2021), “Introduction: explaining pandemic response”, in Greer, S.L., King, E.J., Massard da Fonseca, E. and Perlata-Santos, A. (Eds), Coronavirus Politics, University of Michigan Press.

[ref018] Indelningskommittén (2018), Myndighetsgemensam Indelning – Samverkan På Regional Nivå (SOU 2018:10), Stockholm, available at: https://www.regeringen.se/rattsliga-dokument/statens-offentliga-utredningar/2018/03/sou-201810/

[ref019] Kuhlmann, S., Hellström, M., Ramberg, U. and Reiter, R. (2021), “Tracing divergence in crisis governance: responses to the COVID-19 pandemic in France, Germany and Sweden compared”, International Review of Administrative Sciences, Vol. 87 No. 3, pp. 556-575, doi: 10.1177/0020852320979359.

[ref020] Kuhn, K. and Morlino, I. (2022), “Decentralisation in times of crisis: asset or liability? The case of Germany and Italy during covid-19”, Swiss Political Science Review, Vol. 28 No. 1, pp. 105-115, doi: 10.1111/spsr.12482. 35924083 PMC8662272

[ref021] Lagreid, P. and Rykkja, L.H. (2015), “Organizing for ‘wicked problems’ – analyzing coordination arrangements in two policy areas: internal security and the welfare administration”, International Journal of Public Sector Management, Vol. 28 No. 6, pp. 475-493, doi: 10.1108/ijpsm-01-2015-0009.

[ref022] Maino, F., Blomqvist, P., Bertinato, L., Bohígas Santasusagna, L., Urbanos Garrido, R.M. and Sergey, S. (2007), “Effects of decentralization and recentralization on political dimensions of health systems”, in Saltman, R.B., Bankauskaite, V. and Vrangbaek, K. (Eds), Decentralization in Healthcare, Open University Press.

[ref023] Martindale, A.M., Pilbeam, C., Mableson, H., Tonkin-Crine, S., Atkinson, P., Borek, A., Lant, S., Gobat, N., Solomon, T. and Sheard, S. (2021), “Perspectives on COVID-19 testing policies and practices: a qualitative study with scientific advisors and NHS health care workers in England”, BMC Public Health, Vol. 21 No. 1, 1216, doi: 10.1186/s12889-021-11285-8. 34167491 PMC8224254

[ref024] Mercer, T.R. and Salit, M. (2021), “Testing at scale during the COVID-19 pandemic”, Nature Reviews Genetics, Vol. 22 No. 7, pp. 415-426, doi: 10.1038/s41576-021-00360-w. PMC8094986 33948037

[ref025] OECD (2020), “The territorial impact of COVID-19 : managing the crisis across levels of government”, available at: https://www.oecd.org/coronavirus/policy-responses/the-territorial-impact-of-covid-19-managing-the-crisis-across-levels-of-government-d3e314e1/

[ref026] Olofsson, T., Mulinari, S., Hedlund, M., Knaggård, Å. and Vilhelmsson, A. (2022), “The making of a Swedish strategy: how organizational culture shaped the Public Health Agency's pandemic response”, Qualitative Research in Health, Vol. 2, 100082, doi: 10.1016/j.ssmqr.2022.100082. 35434698 PMC9006404

[ref027] O'Flynn, J. (2013), “Crossing boundaries: the fundamental questions in public management and policy”, in O'Flynn, J., Blackman, D. and Halligan, J. (Eds), Crossing Boundaries in Public Management and Policy: the International Experience, Routledge, London, pp. 11-44.

[ref028] Paquet, M. and Schertzer, R. (2020), “Covid-19 as a complex intergovernmental problem”, Canadian Journal of Political Science, Vol. 53 No. 2, pp. 343-347, doi: 10.1017/s0008423920000281.

[ref029] Peeling, R.W. and Sia, S.K. (2023), “Lessons from COVID-19 for improving diagnostic access in future pandemics”, Lab on a Chip, Vol. 23 No. 5, pp. 1376-1388, doi: 10.1039/d2lc00662f. 36629022

[ref030] Peters, B.G. (2018), “The challenge of policy coordination”, Policy Design and Practice, Vol. 1 No. 1, pp. 1-11, doi: 10.1080/25741292.2018.1437946.

[ref031] Pierre, J. (2020), “Nudges against pandemics: sweden's COVID-19 containment strategy in perspective”, Policy and Society, Vol. 39 No. 3, pp. 478-493, doi: 10.1080/14494035.2020.1783787. 35039732 PMC8754703

[ref044] Public Health Agency of Sweden (2020), “Nationell strategi för diagnostik av covid-19”, Version 1, Stockholm.

[ref032] Saunes, I.S., Vrangbæk, K., Byrkjeflot, H., Jervelund, S.S., Birk, H.O., Tynkkynen, L.K., Keskimäki, I., Sigurgeirsdóttir, S., Janlöv, N., Ramsberg, J., Hernández-Quevedo, C., Merkur, S., Sagan, A. and Karanikolos, M. (2021), “Nordic responses to Covid-19: governance and policy measures in the early phases of the pandemic”, Health Policy, Vol. 126 No. 5, pp. 418-426, doi: 10.1016/j.healthpol.2021.08.011. 34629202 PMC8418563

[ref033] Socialdepartementet (2020a), “Uppdrag om att skyndsamt utöka antalet tester for covid-19”, Stockholm, available at: https://www.regeringen.se/regeringsuppdrag/2020/04/uppdrag-om-att-skyndsamt-utoka-antalet-tester-for-covid-19/

[ref034] Socialdepartementet (2020b), Uppdrag att på nationell nivå säkerställa flöden för storskalig testning för Covid-19, Stockholm, available at: https://www.regeringen.se/regeringsuppdrag/2020/06/uppdrag-att-pa-nationell-niva-sakerstalla-floden-for-storskalig-testning-for-covid-19/

[ref035] Sparf, J. (2022), “Norwegian corporatism: a centripetal national response to the pandemic”, in Zahariadis, N., Petridou, E., Exadaktylos, T. and Sparf, J. (Eds), Policy Styles and Trust in the Age of Pandemics: Global Threat, National Responses, pp. 119-133.

[ref036] Statskontoret (2020), Förvaltningsmodellen under Coronapandemin, Stockholm, available at: https://www.statskontoret.se/globalassets/publikationer/2020/oos41_coronapandemin.pdf

[ref037] Stephenson, P. (2013), “Twenty years of multi-level governance: ‘where does it come from? What is it? Where is it going?’”, Journal of European Public Policy, Vol. 20 No. 6, pp. 817-837, doi: 10.1080/13501763.2013.781818.

[ref038] Vrangbæk, K. (2007), “Towards a typology for decentralization in health care”, in Saltman, R.B., Bankauskaite, V. and Vrangbaek, K. (Eds), Decentralization in Healthcare, Open University Press.

[ref039] WHO (2020), “Laboratory testing strategy recommendations for COVID-19”, available at: https://apps.who.int/iris/bitstream/handle/10665/331509/WHO-COVID-19-lab_testing-2020.1-eng.pdf

[ref042] Zahariadis, N., Petridou, E., Exadaktylos, T. and Sparf, J. (2022), “The Politics of National Responses to the COVID-19 Pandemic”, in Zahariadis, N., Petridou, E., Exadaktylos, T. and Sparf, J. (Eds), Policy Styles and Trust in the Age of Pandemics, Routledge, pp. 3-16.

[ref041] Zahariadis, N., Petridou, E., Exadaktylos, T. and Sparf, J. (2023), “Policy styles and political trust in Europe's national responses to the COVID-19 crisis”, Policy Studies, Vol. 44 No. 1, pp. 46-67, doi: 10.1080/01442872.2021.2019211.

